# Interventions to Relieve the Burden on Informal Caregivers of Older People with Dementia: A Scoping Review

**DOI:** 10.3390/nursrep14030187

**Published:** 2024-09-21

**Authors:** Celia Encinas-Monge, Sergio Hidalgo-Fuentes, Elena Cejalvo, Manuel Martí-Vilar

**Affiliations:** 1 Departamento de Psicología Básica, Universitat de València, Avgda. Blasco Ibáñez 21, 46010 Valencia, Spain; cenmon@alumni.uv.es (C.E.-M.); ecehe@alumni.uv.es (E.C.); 2 Departamento de Psicología y Salud, Facultad de Ciencias de la Salud y de la Educación, Universidad a Distancia de Madrid (UDIMA), Vía de Servicio A-6, 15, Collado Villalba, 28400 Madrid, Spain

**Keywords:** burden, caregivers, dementia, intervention

## Abstract

Dementia increases dependence in older adults and decreases their quality of life and that of their family members. These family members often take on the responsibility of caregiving and suffer from burden and health deterioration due to facing various stressors. The aim is to verify the effectiveness of existing interventions aimed at relieving the burden and stress of informal caregivers of older people with dementia. A scoping review was conducted by consulting the Web of Science, Scopus, ProQuest, and PubMed databases, following the guidelines of the PRISMA 2020 Statement. The review protocol has been registered in PROSPERO under number CRD42024558609. Twenty-six articles met the inclusion criteria and were reviewed, studying the type of intervention design, the sample size of caregivers and their main characteristics, the duration and follow-up, and the variables investigated with their respective measurement instruments. The analysis of the different studies showed that the most developed types of interventions are psychoeducational and cognitive-behavioral therapies, both individual and group. These interventions were mainly effective in reducing the burden and depressive symptoms of caregivers. It is considered advisable to implement more randomized controlled trials for further research, because as the number of caregivers increases, so does the need for affordable and effective interventions.

## 1. Introduction

Recently, the high rate of population ageing has been well documented due to increased life expectancy and declining birth rates. It is estimated that by 2050, 22% of the world’s population will be over 60 years of age [1]. Older adults are experiencing a significant increase in multimorbidity, particularly neuropsychiatric disorders [2]. Among these disorders, dementia is one of the most prevalent and disabling, causing not only disability and dependency for those affected but also high economic costs for individuals, their carers, and society [3]. Dementia is a leading cause of dependence and disability among older adults, affecting more than 55 million people worldwide. Approximately 10 million new cases are reported each year [1]. The relationship between caregiver burden and depression is complex and multifaceted, and it has been extensively studied in the literature. Caregivers of dependent individuals, especially those caring for patients with chronic or degenerative diseases, face significant emotional and physical strain. This burden, which often includes managing daily activities, administering medications, and providing emotional support, frequently lead to a state of exhaustion. Between 40% and 70% of these caregivers meet the diagnostic criteria for major depressive disorder, highlighting the high prevalence of depression in this population. The dependence of the patient on activities of daily living (ADLs) is one of the main factors contributing to caregivers’ psychological distress. This dependence can increase the sense of responsibility, stress, and loss of control, which in turn heightens the risk of developing depressive symptoms. According to the study by Pérez-Mármol et al. (2018), a lack of social support and financial strain can further exacerbate the situation, intensifying feelings of burden and hopelessness in caregivers [4]. In summary, the interplay between caregiver burden and depression is a complex cycle where various individual, social, and contextual factors play a crucial role in a caregiver’s mental health.

Therefore, it is necessary to study not only the diseases but also the physical, mental, and social well-being of the caregivers. The projected increase in dementia cases and the corresponding rise in the number of family members becoming caregivers are closely interconnected. A recent study estimates that dementia cases will increase by 166% by 2050, with around 152 million people expected to suffer from the disease [5]. With the prevalence of dementia in those aged 65 and over estimated to be between 4% and 9% [6], this surge will likely lead to a significant rise in the number of family members taking on caregiving roles. Informal caregivers, who already experience some of the highest rates of depression—between 40% and 70% meeting the criteria for major depressive disorder—will face increased pressure as more individuals become dependent on them for daily living activities [5]. Therefore, it is crucial to study not only the diseases but also the physical, mental, and social well-being of caregivers, as their roles will become increasingly demanding and essential in the coming years.

As a consequence of caring for people with dementia, caregivers’ health deteriorates because they face many stressors associated with the burden. One of the most widely used models to explain burden is that of Pearlin et al. (1990), which synthesises the relationships between caregiver background (social and economic characteristics, history, and relationships) and burden and lack of support [7]. It also considers primary stressors (cognitive impairment and behavioural problems of the person with dementia) and secondary stressors (psychological distress) and mediators (coping and social support). Finally, it considers the impact of stress on the health of informal carers.

There are several formal resources that aim to reduce caregiver burden. Family respite is the most widely used and studied strategy as a stress mediator, as it aims to provide caregivers with a break to engage in other activities [8]. It is provided when a dependent person is under the care of a family member who is taking over their care. The Home Help Service (HHS) is one of the most important services that supports families by taking care of the patient’s daily needs and increasing their autonomy. Day care centres are also an important service, where people can stay for a few hours and be looked after by professional carers. In addition to the formal support services mentioned above, it is important to highlight other existing tools to support informal carers, such as cognitive behavioural therapy and psychoeducational programmes. In psychoeducational programmes, psychologists teach caregivers how to manage stress and cope with everyday situations. Psychoeducational programmes often include training in communication skills, problem-solving, and managing the patient’s behaviour, which enables caregivers not only to respond more effectively to the needs of the person with dementia but also to maintain their own emotional balance. In addition, these programmes can provide a space for caregivers to share experiences and receive mutual support, which can be invaluable in reducing the sense of isolation and overload that often accompanies this work [9,10,11]. Cognitive behavioural therapy is a psychological therapy that aims to change caregivers’ false beliefs and thoughts about caregiving and motivate them to engage in rewarding activities. According to García-Cardoza et al. (2018), this type of therapy has positive effects in reducing the burden on the primary caregiver [12]. Most interventions do not only target the burden of caregivers but also aim to improve their physical and mental health in different domains (stress, depression, well-being, burden, mood, quality of life, etc.). In conclusion, caregivers could benefit from psychoeducational programmes and cognitive behavioural therapies, which would have a positive impact on the person with dementia. However, the evidence on such interventions is limited.

It is essential to emphasise the need to study, develop, and implement global interventions and programmes to reduce the burden on caregivers, and to increase resources to promote research, prevention, early diagnosis, and multidisciplinary approaches. These efforts will help to reduce the health, economic, and, in particular, social and family burdens of dementia. The importance of this review lies in the need to find effective ways to support informal carers of people with dementia. This disorder has significant consequences for both the person with dementia and their carers, as well as for society as a whole. A response to this issue is essential given the increasing prevalence of dementia and the growing role of carers. While psychological tools to reduce depression and improve the quality of life for unpaid family caregivers are undeniably valuable, they risk being mere band-aids if they do not address the deeper systemic issues within our healthcare and social assistance frameworks. The increasing reliance on family care, rather than investing in publicly available care, highlights significant policy failures. It is crucial to recognize that while these psychological interventions are essential for alleviating the immediate burden on caregivers, they must be accompanied by broader efforts to reform and strengthen healthcare and social support systems.

The aim is to review the effectiveness of interventions to date aimed at reducing the burden and stress of informal carers of older people with dementia, and to identify tools that can improve this situation. Therefore, the research question of this study is as follows How do different burden reduction interventions affect informal caregivers of older people with dementia, compared with standard care or no intervention, in terms of reducing depression and burden and improving caregivers’ well-being and quality of life?

## 2. Materials and Methods

### 2.1. Search Strategy

The protocol for this scoping review was registered’ on PROSPERO (CRD42024558609) confirming that the study was not duplicated and reducing the risk of selective outcome reporting bias.

For this review, studies were selected based on the following inclusion criteria: (a) articles or studies published in peer-reviewed scientific journals; (b) ’primary research’; (c) primary empirical research focusing on interventions that qualitatively demonstrate caregiver burden among informal caregivers of older individuals with dementia, with a randomized control group, as this method is considered rigorous for ensuring intervention efficacy; (d) completed studies; (e) available studies; (f) interventions developed in any country were permitted; (g) the study sample consisted of family or informal caregivers of older individuals with dementia; and (h) studies focused on analyzing caregiver burden, stress, and anxiety, and methods to mitigate them were included in this review.

The exclusion criteria that were established were as follows: (a) theses, dissertations, letters, and conference abstracts; (b) articles based on other research, narrative or literature reviews, meta-analysis articles, and proposals for intervention projects; (c) research studies that have not been concluded; (d) research studies that do not focus on caregiver burden and assess other non-relevant aspects for the review; (f) scientific articles published in any language other than Spanish or English; (g) interventions conducted solely with patients or with patients and caregivers together; and (h) interventions developed with fewer than 40 participants. The accuracy of study results largely depends on the sample size [13]. Studies with small sample sizes tend to have greater random variability and, therefore, lower precision in estimating effects. Additionally, studies with few participants have limited statistical power, making it difficult to detect real differences between the groups studied [14]. In a meta-analysis, this can be partially mitigated by weighting the effect sizes of studies by the inverse of their variance, but since this is not possible in a review, it was decided not to include studies with small sample sizes to avoid drawing erroneous conclusions.

A search was performed in January 2024 using the databases Web of Science, Scopus, ProQuest, and PubMed. Search terms included “intervention”, “clinical trials”, “trial”, “caregiver burden”, “caregivers”, “burden”, and “dementia”. Boolean operators “AND”, “OR”, and “NEAR” were employed to link terms related to the topic of interest and obtain relevant literature in the study area.

**Figure 1:**
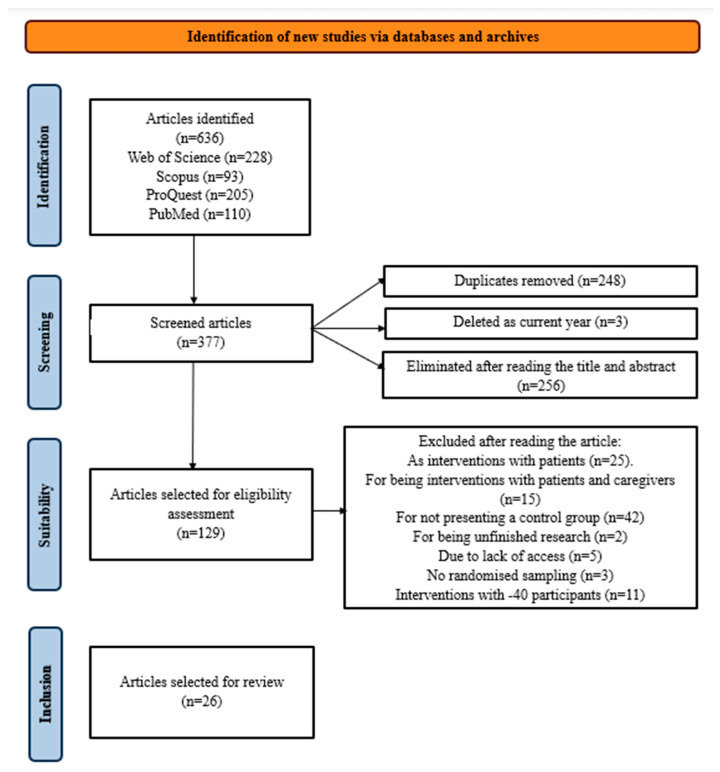
Flow diagram that illustrates the review process.

In the search for research across all databases, a filter was set to include publications up to and including the year 2023. This was done to prevent bias in replicability since the year 2024 had not been completed at the time of the search. Additionally, specific filters were applied within each database’s advanced search options to streamline the selection of pertinent research for this review. In Web of Science, the sole filter applied was to include only scientific articles and clinical trials. In Scopus, due to its extensive filter options, the search was limited to documents categorized as articles, with a finalized publication status, and falling within specified disciplines (medicine, nursing, psychology, social sciences, neuroscience, and health professions). For ProQuest, the search was confined to articles published in peer-reviewed scientific journals. Lastly, in PubMed, the chosen search filter was for article types classified as either clinical trials or randomized controlled trials. The flow diagram (Figure 1) illustrates the review process.

### 2.2. Coding and Data Extraction

Coding and data extraction are important to capture study characteristics that may influence outcomes. Moderator variables recorded included extrinsic factors such as study year and methodological characteristics such as design, intervention type, and sample size. Substantive characteristics related to the subject of the study were also documented. Given the focus on analysing the effectiveness of interventions, it is important to note treatment specifics such as intervention duration and follow-up. Similarly, construct variables were recorded to identify the concept being studied in relation to informal caregiver burden, specifically the study variables (whether the intervention targeted caregiver burden or other related aspects). Outcome variables were also recorded to capture the effectiveness of the intervention (outcomes achieved). In conclusion, the previously developed categories were used to systematically analyse the content of the articles in the review. It is important to note that data were expressed as mean ± standard deviation. Information and data from each selected study were transferred to the Results section, including the following relevant details: (1) study (authors and year), (2) design, (3) intervention type, (4) sample size, (5) intervention duration (format, sessions...) and follow-up, (6) study variables (depression, distress, stress...), (7) measurement instruments, and (8) results obtained. The quality of the included studies, summarised in Supplementary Table S1, was assessed according to the Classification of the Oxford Centre for Evidence-Based Medicine (2009).

## 3. Results

A total of 26 articles met the criteria for this review. Supplementary Table S2 provides a brief summary of the selected studies, while Supplementary Table S3 provides information on the search process.

### 3.1. Design

The design of 80.8% of the studies (*n* = 21) was a randomised controlled trial, and only (*n* = 5) the studies by Brown et al. (2016), Sepe-Monti et al. (2016), Mavandadi et al. (2017), and Rodríguez et al. (2023) were pilot studies [15,16,17,18], accounting for 19.2%. All selected studies had a randomly assigned control group, which helped to avoid selection bias. Studies without a control group (*n* = 42) were excluded from this review because they are considered to be less rigorous in ensuring effectiveness. Trials with non-random allocation (*n* = 3) were also not included. In total, 84.6% of the interventions (*n* = 22) had two treatment groups, an intervention group and a control group, but 15.4% of the interventions (*n* = 4) had three groups. Of the latter, the studies by Cheng et al. (2016) [19] and Hepburn et al. (2022) [20] had two control groups and one intervention group, while Brewster et al. (2020) [21] had one control group and two intervention groups. The control groups in the studies by Spalding-Wilson et al. (2018) [22], Bravo-Benítez et al. (2021) [23], Hives et al. (2021) [24], and Hepburn et al. (2022) [20] were on a waiting list, meaning that 15.4% did not receive any type of intervention or care. The remaining 84.6% of the control groups received standard or usual care. In terms of the blinding of the randomised trials, only the study by Cheng et al. (2016) [19] stated that it was double-blind, meaning that neither the carer nor the researcher knew which study arm the carer had been allocated to, with the aim of eliminating any bias.

### 3.2. Sample Characteristics

The sample size of the interventions in the review varied from 40 to 317 carers. Articles with a small sample size of between 40 and 80 caregivers accounted for 57.7% of the total (*n* = 15), those with a medium sample size of between 81 and 150 caregivers (*n* = 5) accounted for 19.2%, and the remaining articles (*n* = 6) with a large sample size of between 151 and 317 caregivers accounted for 23.1%.

The relationship to the person with dementia and the age and gender of the caregivers involved in the studies were reported in some articles (*n* = 8) but only in 30.77%. In Chang’s (1999) intervention [25], the entire sample consisted of female caregivers, all wives, with an average age of 66.55 years. In Bravo-Benítez et al.’s (2021) sample [23], the average age of the caregivers was 63.88 years, of whom 21.15% were men and 78.85% were women. In Brown et al.’s (2016) sample [15], 84.2% were female caregivers with an average age of 61.14 years. Spalding-Wilson et al. (2018) and Hives et al. (2021) detailed the relationship of the caregiver to the person with dementia [22,24], with the former noting that 52% were children, 45% were spouses, and 3% were siblings. The latter reported that 50% were children, 46% were spouses, 3% were siblings, and less than 1% were nieces/nephews (gender not specified). In addition, in the sample of Tawfik et al. (2021) [26], 60% were wives and 40% were daughters of the people with dementia. 

Thus, based on the data provided by the aforementioned articles, it can be noted that the average age of the caregivers ranged from 60 to 67 years, and in general, the vast majority of the samples were female caregivers, always exceeding 78% of the sample or reflecting the total. It was also observed that the primary carers of a family member with dementia were mostly spouses, closely followed by children, with a very small proportion being siblings and nieces/nephews, typically women.

### 3.3. Type of Intervention

One of the most commonly used interventions was cognitive behavioural therapy (*n* = 6), accounting for 23.1%. This approach helped caregivers identify problematic caregiving situations, become aware of their thoughts, feelings, and behaviours, and make changes to better cope with daily challenges and reduce their burden, as shown in studies by Williams et al. (2019) [27] and Töpfer et al. (2021) [28]. It also helped carers understand why they think, feel or act the way they do. This type of therapy enabled caregivers to explore coping strategies, as in the case of Chang (1999) [25], and to increase awareness of emotions and communication when interacting with people with dementia, as in Brown et al. (2016) [15]. Cheng et al. (2016) used this therapy to reassess caregiving by focusing on its benefits [19], and Spalding-Wilson et al. (2018) combined cognitive behavioural therapy with other therapeutic techniques such as mindfulness and validation therapy [22].

In total, 50% of the studies included in this review were based on psychoeducation (*n* = 13), which helped family carers to understand their situation and that of the person with dementia they were caring for, strengthen their self-esteem, and implement strategies to achieve their well-being and reduce their burden and depression. This technique also provided caregivers with information about the different behaviours that people with dementia may exhibit, such as agitation, apathy, repetitive behaviour and questions, wandering, refusal to take medication, hallucinations, and tips for dealing with these problems. The authors who used psychoeducation in their interventions were Sanford et al. (2007), Martín-Carrasco et al. (2014), Söylemez et al. (2016), Sepe-Monti et al. (2016), Berwig et al. (2017), Mavandadi et al. (2017), Brewster et al. (2020), Terracciano et al. (2020), Bravo-Benítez et al. (2021), Tawfik et al. (2021), Hepburn et al. (2022), Salehinejad et al. (2022), and Rodríguez et al. (2023) [16,17,18,20,21,26,29,30,31,32,33].

Furthermore, 11.5% of the interventions (*n* = 3) were based on physical activity to address caregiver issues related to caring for a person with dementia, such as stress. Farran et al. (2016), Hives et al. (2021), and Madruga et al. (2021) improved caregivers’ psychological functioning by increasing their self-care and setting physical goals based on their interests [24,34,35], thereby increasing their self-esteem.

A further 7.7% of the interventions were based on alternative techniques to alleviate symptoms of caregiver distress (*n* = 2). For example, Hirano et al. (2016) implemented leisure activities with caregivers [36].

Tanner et al. (2015) and Zwingmann et al. (2018) based their interventions on caregiver support and the management of individualised and comprehensive care through interdisciplinarity [37,38], addressing the needs of caregivers by educating them about dementia and developing skill-building strategies. These studies (*n* = 2) represented 7.7% of the total. In addition to analysing the types of therapies used in interventions with informal carers of people with dementia, it is also important to summarise where these interventions were delivered, whether at the carers’ homes, in specific centres, or online, and how they were delivered, either individually or in groups. In total, 53.9% of the interventions were technological and online (*n* = 14), which means that they were delivered through videos, telephone calls, etc., which means that they were delivered in the caregivers’ homes and individually. Follow-up was conducted by professionals, either in person or telematically. Some of the interventions developed through videos that caregivers watched on a computer were those of Chang (1999) [25], Williams et al. (2019) [27], Hepburn et al. (2022) [20], Salehinejad et al. (2022) [33], and Rodríguez et al. (2023) [18]. The videos reflected coping strategies and actions for challenging situations for carers and information on how to care for a person with dementia. There were also presentations with slides and forums to ask questions. Other authors, such as Töpfer et al. (2021) [28], Berwig et al. (2017) [32], and Mavandadi et al. (2017) [17], delivered their interventions over the phone, providing psychosocial support calls to improve the quality of life of carers by developing their skills. Tanner et al. (2015) implemented their individualised care planning via phone [37], and Sanford et al. (2007) and Brewster et al. (2020) combined the use of phone and video [21,29]. Farran et al. (2016) and Madruga et al. (2021) also implemented individualised exercise interventions for carers in their homes, monitored by tools such as pedometers and supervised by a personal trainer [34,35]. Hirano et al. (2016) implemented individualised leisure activities for caregivers in their homes [36]. The interventions by Söylemez et al. (2016) and Sepe-Monti et al. (2016) did not specify whether they were delivered at the caregiver’s home or elsewhere [16,31], or whether they were individual or group-based. The aerobic exercise programme by Hives et al. (2021) was conducted individually at a YMCA gym [24], with the caregivers using heart rate monitors and recording their workouts on the ActiCloud app so that their trainer could email them a progress report. In total, 34.6% of the papers (*n* = 9) reported that the interventions were delivered in groups and away from the carer’s home. Tawfik et al. (2021) implemented their psychoeducational programme to reduce distress and improve quality of life in a specialised group session setting at the geriatric psychiatry unit of the Psychiatry and Addiction Medicine Hospital, Cairo University [26]; Terracciano et al. (2020) and Bravo-Benítez et al. (2021) implemented their respective psychoeducational programmes in community facilities and settings [23,39]. Interventions by Martín-Carrasco et al. (2014), Brown et al. (2016), Cheng et al. (2016), Zwingmann et al. (2018), and Spalding-Wilson et al. (2018) focused on stress reduction, attention, and benefit-seeking and were conducted in groups [15,19,22,30,39].

### 3.4. Duration and Follow-Up

The duration of interventions delivered to informal carers of people with dementia varied considerably, ranging from 2 days [22] to a year and a half [37]. While most interventions lasted 8 weeks or more, Sepe-Monti et al. (2016) conducted their study over 2 weeks, with 6 weekly sessions of 2 hours each [16]. Hepburn et al. (2022) implemented a 43-day programme consisting of 7 synchronous weekly sessions of 75–90 min, accompanied by 36 short asynchronous video lessons for groups [20]. Terracciano et al. (2020) conducted their research over 6 weeks, approximately 1.5 months, with weekly group sessions lasting 2 h, facilitated by two leaders [39]. The most common length of intervention among authors was 8 weeks (*n* = 5), accounting for 19.2%, and 6 months (*n* = 6), accounting for 23.1% of all studies. Chang (1999) [25], Brown et al. (2016) [15], with weekly group sessions of 1.5 h, Cheng et al. (2016) [19], with one 2-hour session per week, Tawfik et al. (2021) [26], with 8 weekly group sessions of 1 h, and Salehinejad et al. (2022) [33], with 12 sessions, all conducted their caregiver-focused studies over 2 months. Hirano et al. (2016) with 30 min of leisure time 3 times a week [36], Berwig et al. (2017) [32], Brewster et al. (2020) with follow-up calls to intervention and control groups [21], Hives et al. (2021) [24], Töpfer et al. (2021) [28], with 12 structured sessions, and Rodríguez et al. (2023) [18] developed their interventions over 6 months. Bravo-Benítez et al. (2021) lasted 2.5 months [23], Mavandadi et al. (2017)and Williams et al. (2019) [17,27] 3 months, and Martín-Carrasco et al. (2014) 4 months [30]. Sanford et al. (2007) and Söylemez et al. (2016) did not specify the duration of their interventions [29,31].

Several studies also reflected on the follow-up conducted by the researchers at the beginning, during, and after the interventions to investigate and verify the effectiveness and duration of the effects. Sanford et al. (2007), Martín-Carrasco et al. (2014), Tanner et al. (2015), and Söylemez et al. (2016) did not specify the follow-up conducted [29,30,31,37]. Similarly, Berwig et al. (2017) did not specify the follow-up but noted that they conducted 12 individual intervention sessions every two weeks (nine home visits of 1.5 h and three telephone sessions of 1 h) and five structured support group sessions once a month [32]. Bravo-Benítez et al. (2021) did not specify follow-up but mentioned 10 sessions of 1.5 h once a week [23]. In many studies (*n* = 15), follow-up was conducted at the beginning of the intervention. This was observed in studies such as Chang (1999) [25], Cheng et al. (2016) [19], Brown et al. (2016) [15], Farran et al. (2016) [34], Sepe-Monti et al. (2016) [16], Mavandadi et al. (2017) [17], Zwingmann et al. (2018) [39], Williams et al. (2019) [27], Terracciano et al. (2020) [39], Hives et al. (2021) [24], Madruga et al. (2021) [35], Tawfik et al. (2021) [26], Hepburn et al. (2022) [20], Salehinejad et al. (2022) [33], and Rodríguez et al. (2023) [18]. Between the start of the intervention and the second month, only Sepe-Monti et al. (2016) [16], Williams et al. (2019) [27], and Terracciano et al. (2020) [39] conducted some form of follow-up, i.e., 11.5%. Similarly, in Chang (1999) [25], Farran et al. (2016) [34], and Rodríguez et al. (2023) [18], controls were conducted between the third and fourth month of the study, also representing 11.5%. In Farran et al. (2016) [24], data collection was conducted in person at month 6 of the intervention and by telephone at month 9. Follow-up was conducted at the end of the intervention in 34.6% of studies (*n* = 9), including Chang (1999) [25], Brown et al. (2016) [15], Cheng et al. (2016) [19], Mavandadi et al. (2017) [17], Williams et al. (2019) [27], Rodríguez et al. (2023) [18], Madruga et al. (2021) [35], Farran et al. (2016) [34], and Salehinejad et al. (2022) [33]. In 42.3% of the studies (*n* = 11), follow-up was performed after the intervention. Terracciano et al. (2020) and Tawfik et al. (2021) reported conducting follow-up after the study without specifying when [26,39], Hives et al. (2021) the following week [24], and Spalding-Wilson et al. (2018) one month later [22]. Follow-up was conducted 3 months after the intervention by Spalding-Wilson et al. (2018) [22], Hepburn et al. (2022) [20], Chang (1999) [25], Brown et al. (2016) [15], and Mavandadi et al. (2017) [17]. Spalding-Wilson et al. (2018) [22], Sepe-Monti et al. (2016) [16], and Hepburn et al. (2022) [20] followed up 6 months after the intervention. In addition, Hepburn et al. (2022) conducted follow-up at 9 months and 1 year [20]. Zwingmann et al. (2018) conducted follow-up at 1 year [38], and Töpfer et al. (2021) at 2.5 years [28].

On the other hand, 42.3% of authors reported dropouts from the programme by caregivers (*n* = 11), which does not help to assess treatment adherence, interest in the intervention, or effectiveness. Hives et al. (2021) reported seven dropouts [24], and Hirano et al. (2016) noted ten [36], without differentiating whether the caregivers who dropped out were from the control or intervention group. Terracciano et al. (2020) reported 5 dropouts after the intervention and 16 after 6 weeks [39]. Spalding-Wilson et al. (2018) reported that 91.5% of caregivers completed the intervention [22], Berwig et al. (2017) reported that 81% completed more than 10 intervention sessions [32], and Hepburn et al. (2022) reported that 14 caregivers from the intervention group, 15 from a control group, and 8 from another group completed the study [20]. Sanford et al. (2007) reported 6 dropouts in the intervention group [29], Sepe-Monti et al. (2016) reported 25 dropouts in the intervention group and 37 in the control group [16], and Tawfik et al. (2021) reported 5 dropouts in both groups [26]. Mavandadi et al. (2017) reported 6 dropouts in the intervention group and 12 in the control group [17], while Rodríguez et al. (2023) reported 3 dropouts in the intervention group and 5 in the control group [18].

### 3.5. Study Variables and Measurement Instruments

The variable most often studied in the various studies was burden, which refers to the emotional and physical exhaustion experienced by people who spend a significant amount of time caring for a dependent person or, in the case of the reviewed documents, caring for someone with dementia. Most of the interventions investigated the variable of burden directly or indirectly, and to measure it, the Zarit Burden Interview (ZBI) was used in 76.9% of the documents (*n* = 20). Its reliability was reported in nine of these articles, all of which showed a reliability greater than α = 0.85, which is considered quite high. Williams et al. (2019) and Mavandadi et al. (2017) used the Modified Zarit Burden Interview with 12 items [17,27]. In addition, Mavandadi et al. (2017) and Chang (1999) also used the Caregiver Appraisal Lawton [17,25]. In the case of Söylemez et al. (2016) and Sanford et al. (2007), no measurement tool was mentioned [29,31]. In 26.9% of studies (*n* = 7), the Mini-Mental State Examination (MMSE) was used to assess suspected symptoms compatible with dementia, with only one study reporting an internal consistency between α = 0.83 and α = 0.98. Chang (1999) and Spalding-Wilson et al. (2018) also used the Functional Assessment Rating Scale (FARS) [22,25], which measures physical function in activities of daily living; Chang reported a reliability of α = 0.86. Hirano et al. (2016), Mavandadi et al. (2017), and Zwingmann et al. (2018) used various scales and surveys of activities of daily living to detect and diagnose dementia, without indicating their reliability [17,36,38].

Depression, psychological distress, and depressive symptoms or distress in informal carers of people with dementia were examined in 65.4% of the documents (*n* = 17). However, researchers used different instruments to measure this variable. Commonly used instruments included the Center for Epidemiologic Studies Depression Scale (CES-D), which was used in 6 out of 26 interventions, with four studies reporting a reliability greater than α = 0.84. Hirano et al. (2016), Sepe-Monti et al. (2016), Mavandadi et al. (2017), and Terracciano et al. (2020) used the Neuropsychiatric Inventory without reporting reliability [16,17,36,39]. Cheng et al. (2016) used the Hamilton Depression Rating Scale without reporting reliability [19]. Spalding-Wilson et al. (2018) used the Beck Depression Inventory [22]. Zwingmann et al. (2018) and Madruga et al. (2021) used the Geriatric Depression Scale by Yesavage [35,38], with the latter reporting a reliability of α = 0.80. In addition, Chang (1999) used the Brief Symptom Inventory [25], reporting α = 0.93. Brewster et al. (2020) used the PROMIS Depression and Anxiety Scales [21], reporting α = 0.93 and α = 0.92, respectively. Madruga et al. (2021) used the Symptom Checklist with α = NR to assess a wide range of psychological and psychopathological symptoms [35]. Sanford et al. (2007), Martín-Carrasco et al. (2014), Söylemez et al. (2016), Hives et al. (2021), Hepburn et al. (2022), and Rodríguez et al. (2023) also investigated the depression variable [18,20,24,29,30,31].

Quality of life, self-efficacy, and coping with the caregiving situation were examined in 30.8% of the studies (*n* = 8). Chang (1999) studied caregivers’ coping using the Moos Coping Strategies Scale with a reliability of α = 0.77 [25], and Sepe-Monti et al. (2016) used the Coping Orientations to Problems Experienced and the Stress Coping Questionnaire without indicating reliability [16]. Martín-Carrasco et al. (2014) and Cheng et al. (2016) studied quality of life using health surveys [19,30], and Tawfik et al. (2021) used the Quality of Life Questionnaire with α = NR [26].

Stress and anxiety were investigated in 26.9% of the interventions with family caregivers of dementia patients (*n* = 7), for example, by Brown et al. (2016), Sepe-Monti et al. (2016), Berwig et al. (2017), and Brewster et al. (2020) [15,16,21,32]. One of the instruments used to measure stress was the Perceived Stress Scale, chosen by Brown et al. (2016), Spalding-Wilson et al. (2018), Mavandadi et al. (2017), and Hepburn et al. (2022) [15,17,20,22], with Brown and Hepburn reporting reliability greater than α = 0.75. Spalding-Wilson et al. (2018) also used the Beck Anxiety Inventory [22], a useful tool for assessing somatic symptoms of anxiety, without reporting reliability. Mavandadi et al. (2017) and Sepe-Monti et al. (2016) used the State-Trait Anxiety Inventory without reporting internal consistency [16,17].

Other variables and measures have also been investigated on a smaller scale. Brown et al. (2016) studied mood states using the Profile of Mood States [15], reporting α = 0.96 for depressive symptoms, α = 0.92 for fatigue, and α = 0.83 for confusion. Farran et al. (2016) used the Positive and Negative Affect Scale and reported α = 0.91 for caregiver affect [34]. Bravo-Benítez et al. (2021) and Hives et al. (2021) used the Positive Aspects of Caregiving Scale, reporting reliabilities of α = 0.82 and α = 0.92, respectively [23,24]. Williams et al. (2019) examined sleep disturbance using the Pittsburgh Sleep Quality Index [27], reporting α = 0.83, and caregiver competence and mastery using the Caregiver Competence Scale, with a reliability of α = 0.76. Brewster et al. (2020) also measured competence using the Caregiver Competence Scale [21], with α = 0.79, and Martín-Carrasco et al. (2014) measured insomnia using the Anxiety and Insomnia Subscale [30], with α = NR. Terracciano focused on caregiver efficacy and confidence using the Caregiver Self-Efficacy Scale with α = NR [39]. Bravo-Benítez et al. (2021) used several instruments [23], including the Caregiver Grief Scale (α = 0.85), the Resilience Scale (α = 0.90), the Acceptance Questionnaire (α = 0.88), and the Post-Traumatic Growth Inventory (α = 0.95). Brown et al. (2016) also used the Acceptance Questionnaire and reported a reliability of α = 0.86 [15]. Hives et al. (2021) used the UCLA Loneliness Scale (α = 0.86), the Social Provisions Scale (α = 0.89), the Rumination Scale (which assesses repetitive intrusive and premeditated thoughts following a major stressor) with α = 0.93, the Expressive Suppression Subscale of Emotional Regulation (α = 0.61), and the Pearlin Mastery Scale (α = 0.78) [24]. Hepburn et al. (2022) and Mavandadi et al. (2017) also used the Mastery Scale [17,20], with the former reporting a reliability of α = 0.76. Salehinejad et al. (2022) used the Alzheimer’s Disease Knowledge Scale [33], and Rodríguez et al. (2023) used the Usability Scale of the Simplified System [18], both reporting α = NR. In addition, different general health scales, surveys and questionnaires have been conducted, for example by Martín-Carrasco et al. (2014) [30], Brown et al. (2016) [15] reporting α = 0.81, Sepe-Monti et al. (2016) [16], Bravo-Benítez et al. (2021) [23] reporting reliabilities of α = 0.70 to 0.90, and Töpfer et al. (2021) [28] reporting consistencies of α = 0.83 for physical health, α = 0.81 for psychological health, and α = 83 for quality of life. Various self-administered questionnaires were also used to assess carers’ attitudes and intentions, their health, and to evaluate and monitor interventions. In 30.8% of the studies (*n* = 8), the reliability or internal consistency of all the instruments used was measured. In 53.8% of the documents (*n* = 14), the reliability of the measurement tools was not reported and in 15.4% of the studies, the reliability was reported for some tools but not for others.

### 3.6. Results and Effectiveness of Interventions

In 15.4% of studies (*n* = 4), there was no difference or improvement in the group receiving the intervention compared with the usual care group. The study by Martín-Carrasco et al. (2014) found that the intervention was not superior to usual care on any variable [30]. With regard to Tanner et al. (2015) [37], they observed a reduction in unmet needs and time spent by carers providing care, but the variables of burden, depression and quality of life did not differ from the usual care group. Söylemez et al. (2016) showed that the intervention was effective in reducing burden and depression and improving quality of life, but no more than usual care [31]. Mavandadi et al. (2017) found no differences in burden between groups [17].

The vast majority of studies (*n* = 21) investigated the burden and depression of informal carers, 80.8% of all documents. The authors found that both variables decreased in the intervention groups compared with those receiving standard care. Chang (1999) found that depression increased in the control group between weeks 4 and 12, while it remained stable in the intervention group [25]; Sanford et al. (2007) and Cheng et al. (2016) observed improvements in burden and depressive symptoms [19,29]. Hirano et al. (2016), Tawfik et al. (2021), Töpfer et al. (2021), and Salehinejad et al. (2022) expressed a decrease in burden [26,28,33,36], Farran et al. (2016) noted an improvement at 3 months [34], and Berwig et al. (2017) reported a slight decrease in the intervention group and a significant increase in the control group, although it shifted from a large effect to a moderate effect at 3 months [32]. The latter authors also observed improvements in depression, similar to the study by Sepe-Monti et al. (2016) [16]. Terracciano et al. (2020), Hives et al. (2021), and Madruga et al. (2021) found reductions in depressive symptoms and distress [24,35,39]; Rodríguez et al. (2023) showed that distress decreased in the intervention group, although it increased over the months, and psychological symptoms decreased from the start [18]. Spalding-Wilson et al. (2018) showed that burden decreased each month, while depression decreased between the beginning of the intervention and the first month [22]; Zwingmann et al. (2018) reported a decrease in both objective and subjective burden, with the control group showing an increase at 12 months [38]; Williams et al. (2019) showed that higher levels of education were associated with improvements in burden, and depression decreased in the intervention group at 3 months [27]; and Bravo-Benítez et al. (2021) reported an increase in burden between initial evaluations in the control group and a reduction in the intervention group [23]. In the study by Brewster et al. (2020), depressive symptoms and burden decreased in the intervention group, while the control group experienced deterioration in both variables [21]. Hepburn et al. (2022) expressed that depression improved in over 60% of caregivers from baseline to 3 and 6 months, with little or no improvement in one control group and worsening over time in another [20]. Burden decreased over time in the intervention group. Interventions were not always effective in improving burden and depression variables. Hirano et al. (2016) did not reduce depression [36], and Sepe-Monti et al. (2016) did not succeed in reducing the burden of informal caregivers of people with dementia [16].

Other variables such as anxiety, stress, mastery, quality of life, and coping have also been studied. Chang’s (1999) intervention was effective in reducing caregiver anxiety [25], and Brown et al. (2016) reduced stress and improved psychosocial factors [15]. Spalding-Wilson et al. (2018) showed that stress and anxiety decreased between the start of the intervention and the first month, and continued for up to 6 months [22], and Hepburn et al. showed that anxiety decreased in over half of caregivers from baseline to 3 and 6 months [20]. These studies (*n* = 4) investigating stress and anxiety in carers represented 15.4%. Regarding changes in levels of epinephrine, norepinephrine, dopamine, and cortisol in caregivers, Hirano et al. (2016) and Berwig et al. (2017) found no changes [32,36]. The latter also found improvements in somatisation and caregiver reactions. In addition, Sepe-Monti et al. (2016) showed that relatives of caregivers in the intervention group received fewer new prescriptions for neuroleptics over 6 months [16]. In total, 26.9% of the interventions examined mastery (*n* = 7). Mavandadi et al. (2017), Sepe-Monti et al. (2016), Williams et al. (2019), and Brewster et al. (2020) demonstrated improved coping and mastery, and a greater reduction in distress [16,17,21,27]. Hives et al. (2021) also noted mastery improvement in the intervention group [24], and Hepburn et al. (2022) and Salehinejad et al. (2022) showed better reactions to dementia-related behaviors [20,33]. On the other hand, Tawfik et al. (2021) and Töpfer et al. (2021) stated that quality of life for informal caregivers of people with dementia improved due to the interventions [26,28].

## 4. Discussion

The aim of this scoping review was to determine the effectiveness of different interventions aimed at reducing the burden and stress of informal carers of older adults with dementia and to identify tools that can improve this situation. This was achieved by analysing the research studies, different techniques, and programmes used, and assessing their strengths and limitations. The Preferred Reporting Items for Systematic reviews and Meta-Analyses extension for Scoping Reviews (PRISMA-ScR) Checklist was followed in conducting this scoping review (see Supplementary Table S4).

Not all interventions with informal carers of older adults with dementia are the same, nor do they use identical techniques to improve burden, stress, or depression. Cognitive behavioural interventions were used in 23.1% of the included studies (*n* = 6), psychoeducation in 50% (*n* = 13), physical activity in 11.5% (*n* = 3), alternative techniques such as leisure activities, relaxation, and massage in 7.7% (*n* = 2), and individualised and comprehensive care management in 7.7% (*n* = 2). In addition, 53.9% of interventions (*n* = 14) were technological and online, delivered in the carer’s home and usually on an individual basis, while 34.6% were group-based and delivered outside the home (*n* = 9). It should be noted that one study was conducted individually in fitness centres. The majority of the synthesised documents, 80.8%, examined the burden and depression of informal carers (*n* = 21) and found that both variables decreased in the intervention groups compared with the control groups, regardless of the type of intervention. Most cognitive-behavioural interventions reported improvements in burden and reflected benefits in depression. Chang (1999) indicated that depression increased in the control group between weeks 4 and 12, whereas it remained stable in the intervention group [25]. Spalding-Wilson et al. (2018) reported a reduction in depression from baseline to the first month of the programme [22], and Williams et al. (2019) found a reduction in depression in their intervention group after 3 months [27]. Psychoeducational interventions have also reduced distress and depressive symptoms. Berwig et al. (2017) reported reduced burden in the intervention group, which moderated from a large effect to a moderate effect after 3 months [32], and Rodríguez et al. (2023) showed improved burden in the intervention group, although it increased over the months, with a progressive decrease in psychological symptoms [18]. Hepburn et al. (2022) documented that more than 60% of carers experienced an improvement in depression and a gradual reduction in distress over 3 to 6 months [20]. Similarly, interventions based on physical activity, such as those by Hives et al. (2021) and Madruga et al. (2021), led to reductions in burden and depression [24,35]. Farran et al. (2016) observed improvements in these variables after 3 months [34]. In the study by Hirano et al. (2016), based on alternative techniques, burden decreased [36]. Zwingmann et al. (2018), whose programme focused on individualised care management, also reduced both objective and subjective burden [38]. Therefore, the results are generally positive, as most interventions were successful in reducing caregiver burden and improving depression. The study by Zabalegui-Yárnoz et al. (2008) agrees, with statistically significant positive results for burden in 40% of cases and for depression in 90% of cases [40]. This suggests that interventions targeting informal carers are beneficial not only in reducing burden but also in reducing associated factors such as depression.

However, as seen in the various results presented earlier, not all studies indicate when variables start to improve, how long the effects last, or how long it takes for the benefits to fade. Furthermore, interventions are not always effective. Hirano et al. (2016) found that leisure activities at home did not reduce depression [36], and Sepe-Monti et al.‘s (2016) psychoeducational programme did not reduce caregiver burden [16]. Several studies also examined anxiety, stress, or quality of life in informal carers. In total, 15.4% of studies (*n* = 4) reported reductions in stress and anxiety. Spalding-Wilson et al. (2018) and Hepburn et al. (2022) also documented reductions from baseline to 6 months [20,22]. The aforementioned review by Zabalegui-Yárnoz et al. (2008) also showed statistically significant positive outcomes for anxiety in 50% [40].

The follow-up carried out by the researchers to check the effectiveness and duration of the effects varies. Some studies (*n* = 7) report the chronological development of intervention activities as seen in the outcomes, but do not specify when the follow-up takes place. In total, 42.3% of authors reported caregiver dropouts from programmes (*n* = 11), which may help to assess adherence, interest in the intervention, or effectiveness. Two authors reported dropouts in their studies without differentiating between caregiver groups, one researcher reported when dropouts occurred, and five reported how many dropouts occurred in the control and intervention groups. The discrepancy in dropout rates between studies may be due to different sample sizes in the interventions and differences in the number of caregivers in the control and intervention groups. In addition, dropouts may be due to many factors, such as the type of intervention, the caregiver’s condition, lack of adherence and monitoring at follow-up, insufficient self-assessment of improvement, and others. The different outcomes found at follow-up may also be related to differences in the duration of the interventions.

This review begins with the question: How do interventions designed to reduce burden affect informal caregivers of older adults with dementia, compared with standard care or no intervention, in terms of reducing depression and burden and improving caregivers’ well-being and quality of life? It is observed that the vast majority of studies examine caregiver burden and depression, and both are improved in intervention groups compared with control groups, regardless of the type of intervention. Although there is no consensus on which types of intervention are most effective, all show benefits on caregiver burden and depression, as well as improved quality of life or coping mechanisms, thus answering the original question. The improvement in the lives of carers and the new knowledge provided by the interventions developed will indirectly and positively influence the well-being of older adults with dementia.

The present review has several methodological limitations. It is important to note that the findings obtained in this study are mostly descriptive, without the inclusion of meta-analyses that could provide a more robust quantitative assessment of the interventions. This limits the ability of the research to fully answer the question posed.

Another significant methodological limitation is that the selected studies focus predominantly on interventions based on psychotherapeutic and/or psychiatric approaches, such as cognitive behavioural therapy (CBT), mindfulness training, and physical exercise, all of which target the well-being of the individual caregiver. However, these studies do not include interventions that deliberately focus on improving the interaction between patient and caregiver.

There is also little discussion of physiological endpoints, which could clarify assessment issues, and a lack of analysis of deterioration reported by control groups during interventions.

## 5. Conclusions

This review examines several interventions that have been developed to reduce the burden on informal carers of older people with dementia. The analysis of different studies shows efficacy, particularly in reducing burden and depression, and also indicates improvements in other variables such as stress, anxiety, and even quality of life.

The most commonly used interventions to reduce caregiver burden are psychoeducational programmes and cognitive-behavioural therapy, delivered both individually and in groups, either online in the caregiver’s home or in designated community settings. These two methods were not only the most widely used but also the most effective. In addition, interventions based on leisure activities, physical activities, and massage, as well as those supported by individualised and comprehensive care management for carers, although less commonly used, were also beneficial.

As the number of informal carers continues to increase, there is a growing need for affordable, brief, and effective interventions to reduce burden and depression. Furthermore, given the impact of caregiving on people with dementia, it is important to identify non-pharmacological methods to improve caregivers’ quality of life, well-being, and psychological functioning. From a theoretical perspective, this review lays the groundwork for future research aimed at establishing interventions to reduce burden and depression in informal carers.

## Data Availability

No new data were created or analyzed in this study. Data sharing is not applicable to this article.

## Supplementary Materials

The following supporting information can be downloaded at: https://www.mdpi.com/article/10.3390/nursrep14030187/s1. Table S1: Oxford Levels of Evidence Classification (CEBM); Table S2: Synthesis of the articles reviewed; Table S3: Search strategy; Table S4: Preferred Reporting Items for Systematic reviews and Meta-Analyses extension for Scoping Reviews (PRISMA-ScR) Checklist.

## Public Involvement Statement

No public involvement in any aspect of this research.

## Guidelines and Standards Statement

This manuscript was drafted against Preferred Reporting Items for Systematic Reviews (PRISMA).
