# Supplementary material for: Interventions to Relieve the Burden on Informal Caregivers of Older People with Dementia: A Scoping Review
Source: Nurs Rep. 2024 Sep 21;14(3):2535–49. doi: 10.3390/nursrep14030187 (PMC11417853; doi:10.3390/nursrep14030187)
Supplement: Supplementary file 1 [file nursrep-14-00187-s001.zip › nursrep-3140431-supplementary.pdf]

Table S1. Oxford Levels of Evidence Classification (CEBM).

Estudy	**Level of recommendation**	**Evidence level**
Chang (1999) [1]	A	1A
Sanford et al. (2007) [2]	A	1A
Martín et al. (2014) [3]	A	1A
Tanner et al. (2015) [4]	A	1A
Brown et al. (2016) [5]	A	1A
Cheng et al. (2016) [6]	A	1A
Farran et al. (2016) [7]	A	1A
Hirano et al. (2016) [8]	A	1A
Sepe et al. (2016) [9]	A	1A
Söylemez et al. (2016) [10]	A	1A
Berwig et al. (2017) [11]	A	1A
Mavandadi et al. (2017) [12]	A	1A
Spalding et al. (2018) [13]	A	1A
Zwingmann et al. (2018) [14]	A	1A
Williams et al. (2019) [15]	A	1A
Brewster et al. (2020) [16]	A	1A
Szczepańska et al. (2020) [17]	A	1A
Terracciano et al. (2020) [18]	A	1A
Bravo et al. (2021) [19]	A	1A
Hives et al. (2021) [20]	A	1A
Madruga et al. (2021) [21]	A	1A
Tawfik et al. (2021) [22]	A	1A
Töpfer et al. (2021) [23]	A	1A
Hepburn et al. (2022) [24]	A	1A
Salehinejad et al. (2022) [25]	A	1A
Rodríguez et al. (2023) [26]	A	1A

Table S2. Synthesis of the articles reviewed.

Study	Desing	Intervention	Sample size	Duration I and S	Variables	Instruments	Outcomes
Chang (1999) [1]	RCT	I cognitive-behavioural video	65C<br>(wives, average age 66.55 years)<br>- IG: 31<br>- CG: 34 attention-only phone calls	8 weeks<br>S: start and at 4, 8 and 12 weeks	- Coping strategies<br>- Burden<br>- Satisfaction<br>- Physical and emotional health	- Moos Coping Strategies (α=0.77)<br>- Caregiver Appraisal Lawton (burden α=0.79 and satisfaction α=0.72)<br>- Brief Symptom Inventory (α=0.93)<br>- Form Force I<br>- S Carga Zarit (α=0.91)<br>- Mini Mental State Examination (α=0.83 a 0.98)	Both groups: reduction in anxiety and satisfaction (related to chronic dementia).CG increased depression between weeks 4 and 12, and IG stable over time.<br>Depression and anxiety greater change.

						- S Hutton Functional Qualification (α=0.86)	
Sanford et al. (2007) [2]	RCT	I technology-based psychoeducation	46 C<br>- IG: 23<br>- CG: 23 I information only	Abandonments: 6GI	- Burden<br>- Depression	NR	IG: the burden decreased, and those with high depression at baseline experienced improvement.
Martín et al. (2014) [3]	RCT	I psychoeducational group	238 C<br>- IG: 115<br>- CG: 123	4 months	- Burden<br>- Psychological distress<br>- Quality of life	- S Zarit (α=NR)<br>- Health Surveys<br>- General Health Questionnaire (α=NR)<br>- Anxiety and Insomnia subscale (α=NR)	IG: scores on the measuring instruments did not show any differences.
Tanner et al. (2015) [4]	RCT	I coordination. Multi-component,	289 C<br>- IG: 106 referral and linkage to	1 year and a half	- Unmet care needs	- Mini Mental State Examination (α=NR)	

		community-based, individualised care planning and interdisciplinary S	dementia services, provision of education and skills strategies<br>- CG: 183		- Burden, quality of life and depression		Both groups: C's unmet needs decreased.<br>Burden, depression and quality of life: no differences.<br>IG: reduction in hours spent on care by Cs.
Brown et al. (2016) [5]	RCT, pilot	Tailored mindfulness-based stress reduction programme	40 C<br>(84.2% female and average age 61.14 years old)<br>- IG: 24<br>- CG: 16 standard social support.	8 weeks<br>Weekly group classes of 1.5 to 2 hours per week<br>F: start, end, and at 3 months	-Stress and burden<br>-Mental health<br>- Improved care<br>- Experiential avoidance	- S Perceived Stress Cohen (α=0.75)<br>- Questionnaire II Acceptance and Action (α=0.86)<br>- Profile Mood States (depressive symptoms α=0.96,	Both groups: reduced stress and altered mood. IG: lower levels.S 3 months: both groups psychosocial improvements.<br>No difference in change in diurnal salivary cortisol

					- Relationship quality	fatigue α=0.92, confusion α=0.83 ...)<br>- Brief Mental and Physical Health Survey (α=0.81)<br>- S Zarit (α=0.89)<br>- S Mutuality (α=0.94)<br>- Saliva collection (6 times/day)	response during the study.
Söylemez et al. (2016) [10]	RCT	Progressively lowered stress threshold model	65 C<br>- IG<br>- CG	NR	- Burden<br>- Depressive symptoms<br>- Quality of life	NR	Both groups: equally effective in reducing burden, depression and increasing quality of life.<br>IG: improve variables in the F.
Cheng et al. (2016) [6]	RCT	I profit-focused reassessment	129 C<br>- IG	8 weeks	- Depression<br>- Overload	- S Hamilton depression (α=NR)	IG: improvements in depressive symptoms

		or profit-seeking	- CG1: simplified psychoeducation with lectures<br>- CG2: standard	1 session of 2h each week<br>F: start and end	- Psychological well-being<br>- Positive self-efficacy and achievement	- S Zarit (α=NR)<br>- Short Health Surveys	and burden compared to CG1 and CG2. More significant improvement in depressive symptoms.
Farran et al. (2016) [7]	RCT	I Enhanced individualised physical activity	211 C<br>- IG: 106<br>- CG: 105 I development of standard skills	1 year<br>F: initial home visit and periodic calls.Data collected: in person baseline, 6 and 12 months; by phone 3 and 9 months	- Subjective burden<br>- Depressive symptoms<br>- Positive affect	- S Zarit (α=0.87)<br>- S Depression Epidemiological Studies Centre (α=0.92)<br>- S Positive and Negative Affect (α=0.91)<br>- Mini Mental State Examination (α=NR)	IG: increased overall and total moderate physical activity and improved positive affect from baseline to 6 and 12 months compared to CG.<br>IG: improved load at 3 months, but no effect on depressive symptoms.

Hirano et al. (2016) [8]	RCT	Leisure activities at home	42 C<br>- IG: 21<br>- CG: 21 normal care activities	6 months.<br>Leisure activities 30 minutes 3 times a week<br>Dropouts: 10	-Attention load<br>-Stress hormone levels	- S Zarit (α=NR)<br>- Activity of Daily Living Survey<br>- Mini Mental State Examination (α=NR)<br>- Neuropsychiatric Inventory (α=NR)	IG: load decreased.<br>CG: no difference in load.<br>Both groups: no change in adrenaline, noradrenaline, dopamine and cortisol levels.
Sepe et al. (2016) [9]	RCT, pilot	Psychoeducation programmes	164 C<br>- IG: 80<br>- CG:84	2 weeks<br>6 weekly sessions of 2h<br>S: before, after 2 weeks and 6 months after completion<br>Dropouts: (25 IG and 37 CG)	- Burden<br>- Distress<br>- Psychological symptoms<br>- Coping strategies adopted	-S Zarit (α=NR)<br>- S Coping Orientations (α=NR)<br>- Neuropsychiatric Inventory (α=NR)<br>- S Brief Health Survey (α=NR)	IG: better coping strategies and less anxious and depressed than CG. IG: burden was not reduced. IG C patients received fewer neuroleptic prescriptions than GC, during the 6 months F. Apathy symptom improved more.

						- E Depression Epidemiological Studies (α=NR)<br>- Stress Coping Questionnaire (α=NR)<br>- Mini Mental State Examination (α=NR)<br>- State-Trait Anxiety Inventory (α=NR)	
Berwig et al. (2017) [11]	RCT	I multicomponent individualised, psycho-educational and evidence-based skills training	92 C<br>- IG: 47(15 men, 32 women, mean age 72.3 years)<br>- CG: 45 (16 men, 29 women, mean age 74 years)	6 months.12 fortnightly individual sessions (9 at home of 1.5 hours and 3 telephone sessions of 1 hour).5	- Burden<br>- Challenging behaviour<br>- Stress<br>- Caring capacity - Caring capacity	- S Zarit (α=0.91)<br>- Mini Mental State Examination (α=NR)<br>- Health Questionnaires	Burden decreased a little in IG and increased a lot in CG. Effect decreased from large to moderate in F 3 months. Improved somatization, health-

				structured telephone support group sessions (1 time per month)<br>81% completed more than 10 sessions	- Social support<br>- Physical and emotional well-being		related quality of life and reactions.GI: more depressed, more somatization, lower psychological health-related quality of life and stronger reaction to challenging actions.19 IG C's and 7 CG C's visited support group in I.Both groups: = adrenaline, noradrenaline, dopamine and cortisol levels.<br>.
Mavandadi et al. (2017) [12]	RCT, pilot	Collaborative dementia care management	75 C<br>- IC: 37<br>- CG: 37		- Distress	- S Activities of Daily Living Katz (α=NR)	IG: greater reductions in distress and

		programme by telephone		3 months<br>Minimum of 3 calls<br>F: start, after 3 and 6 months<br>Dropouts: 6IG and 12CG	- Mastery and coping<br>- Burden	- Neuropsychiatric Inventory (α=NR)<br>- Sub E Zarit (α=NR)<br>- Caregiver Appraisal Lawton (α=NR)<br>- S Perceived Stress (α=NR)<br>- State-Trait Anxiety Questionnaire (α=NR)<br>- S Pearlin Domain (α=NR)	improvements in coping and mastery. No differences between groups in symptom burden, frequency or severity.
Spalding et al. (2018) [13]	RCT	I with exercises and improvisation techniques to improve self-care and communicatio	104 C<br>(52% children, 45% spouses and 3% siblings)<br>- IG: 62 I manual on mindfulness, validation and	2 days<br>F: at 1 month and at 3 and 6 months<br>91.5% completed I	- Burden<br>- Stress<br>- Anxiety<br>- Depression	- S Zarit (α=NR)<br>- S FAST Functional Rating (α=NR)<br>- S Perceived Stress (α=NR)	IG: reduction in perceived stress up to 6 months later, and respondents found it helpful in controlling behaviours.

		n, practising skills.	behavioural management<br>- CG: 42 waiting list			- Beck Depression Inventory II (α=NR)<br>- Beck Anxiety Inventory (α=NR)	Burden decreased each month.<br>Stress, anxiety and depression decreased between baseline and first month.
Zwingmann et al. (2018) [14]	RCT	Testing the effectiveness of dementia care management on multifaceted burden	317 C<br>- IG: 226<br>-CG: 91	1 year<br>F: start and 12 months later	- Burden<br>- Health problems	- Mini Mental State Examination (α=NR)<br>- S Bayer Activities of Daily Living (α=NR)<br>- S Yesavage Geriatric Depression (α=NR)<br>- Berlin Burden Inventory (α=NR)	IG: decreased objective burden (emotional support), subjective burden due to behavioural change (cognition, aggression and depression) and due to conflicts in caring (financial losses) compared to CG, showed increased burden in F 12 months.
Williams et al. (2019) [15]	RCT	Telehealth technology support (experts review videos	83 C<br>- IG: 42 recordings C send of challenging care	3 months<br>Weekly individual sessions	- Burden<br>- Depression	- Modified Zarit S (α=0.88)<br>- Sense of Competence	Both groups: higher education level was associated with greater improvements in

		and provide individualized assistance)	situations and receive feedback<br>- CG: 41 telephone support	F: start, at 1 month and at 3 months	- Sleep disorders<br>- Competition	Questionnaire (α=0.76)<br>- S Depression Centre Epidemiological Studies (α=0.84 to 0.90)<br>- Pittsburgh Sleep Quality Index (α=0.83)<br>- Modified Desire to Institutionalise Scale (α=0.69 to 0.77)	burden, competence and reaction to behavioural symptoms.<br>IG: greater reductions in depression and gains in competence after 3 months compared to CG.
Brewster et al. (2020) [16]	RCT	I psychoeducational	142 C<br>- IG1: 41 ◎ 6 group sessions of 2h, during consecutive weeks.<br>- IG2: 48 ◎ I plus exercise. Moderate intensity walking	6 months<br>F IG1: calls 1/month<br>F IG2: fortnightly calls for 1-month, weekly calls for 1	- Depressive symptoms<br>- Anxiety<br>- Burden<br>- Dominance	- S Zarit (α=0.92)<br>- S PROMIS Depression (α=0.93)<br>S PROMIS Anxiety (α=0.92)<br>- S Care Competence (α=0.79)	IG1 and IG2: reduction of depressive symptoms and improvement in mastery at 6 months.<br>IG1: reduction in anxiety.<br>CG: worsened C load.

			30 min, 5 days/week and resistance training sessions 30 min, 2 days/week.<br>CG: 53	month and bimonthly calls for the rest of the time.<br>F CG: telephone call control attention after 4-5 months			
Szczepańska et al. (2020) [17]	RCT, pilot	I of non-pharmacological forms of therapy	45 C<br><br>(40 women and 5 men)<br><br>- IG1: 15 relaxing massages<br><br>- IG2: 15 group relaxation<br>- CG: 15	1 year<br><br>Massage: 10 sessions of 45 min, 5 times per week<br>Relaxation: 45 min sessions, Monday-Friday for 2 consecutive weeks	- Burden<br>- Emotional state	- S Social Support Berlin (α=0.71 to 0.90)<br><br>- S Zarit (α=NR)<br><br>- Beck Depression Inventory (α=0.95)<br><br>- S Life Satisfaction (α=NR)<br>- Information Questionnaire	IG1 and IG2: reduction of depressive symptoms and improvement in mastery at 6 months.<br><br>IG1: reduction in anxiety.<br><br>CG: worsened C load.<br><br>IG1 and IG2: effective in reducing burden

							and improving psychophysical condition.<br>IG1: improved load and mood.<br>IG2: no effect on load, but improved mood and well-being.<br>CG: deterioration in mental state and load during I.
Terracciano et al. (2020) [18]	RCT	I psycho-educational tools	60 C<br>- IG: 24<br>- CG: 36	6 weeks<br>Weekly group lessons of 2h, facilitated by 2 leaders<br>F: start, after I and after 6 weeks	- Burden<br>- Depressive symptoms<br>- Self-efficacy and confidence<br>- Self-care	- S Zarit (α=NR)<br>- Cohen Agitation Inventory (α=NR)<br>- S Depression Centre for Epidemiological Studies (α=NR)<br>- S Self-efficacy Caregiving (α=NR)	IG: reduced burden and depressive symptoms; and increased confidence. No benefit for behavioural and psychological symptoms. Participants rated high on I and low dropout,

				Drop-outs: 5 after I and 16 after 6 weeks	- Emotional distress	- Neuropsychiatric Inventory (α=NR)<br>- Satisfaction Questionnaire	94 % C completed at least 4/6 classes.
Bravo et al. (2021) [19]	RCT	I to ameliorate bereavement-related symptoms	52 C<br>(mean age 63.88 years, 21.15% male and 78.85% female)<br>- IG: 27<br>(3 groups of 5 C and 2 groups of 6)<br>- CG: 25 waiting list	2 and a half months<br>10 I sessions of 1.5 hours, 1 time per week	-Overload<br>- Grief<br>- Resilience<br>- Experiential avoidance<br>- Quality of life<br>- Benefits of care	- S Zarit (α=0.91)<br>- S Caregiver Grief (α=0.85)<br>- S Resilience (α=0.90)<br>- Acceptance Questionnaire (α=0.88)<br>- Posttraumatic Growth Inventory (α=0.95)<br>- Positive Aspects Caregiving (α=0.82)<br>- Health Survey (α=0.70 to 0.90)	Both groups: decrease in despair.<br>Load increased between 1st and 2nd assessment in CG, and decreased in IG.<br>Resilience decreased between 1st and 2nd assessment in CG and increased in IG.<br>IG: avoidance decreased.<br>Positive aspects of care and health increased in IG.

Hives et al. (2021) [20]	RCT	Aerobic exercise programme on psychological functioning	68 C<br><br>(50% children, 46% spouses, 3% siblings and less than 1% nieces and nephews)<br><br>- IG: 34<br><br>- CG: 34 waiting list	6 months<br><br>F: start and week after completion Dropouts: 7	- Load<br><br>- Dominance<br><br>- Depression	- S Positive Aspects Care (α=0.92)<br><br>- S Zarit (α=0.85)<br><br>- S Patient Health Questionnaire (α=0.81)<br><br>- S UCLA Loneliness (α=0.86)<br><br>- S Social Provisions (α=0.89)<br><br>- S Rumination (α=0.93)<br><br>- Sub E Expressive Suppression of Emotional Regulation Questionnaire (α=0.61)<br><br>- S Pearlin Domain (α=0.78)	IG: decreased depressive symptomatology, compared to GC.<br><br>I revealed reductions in C load, as well as increases in IG domain compared to CG.

						- Life Orientation Test (α=0.71)	
Madruga et al. (2021) [21]	RCT	Home-based physical exercise programme on mental health	48 C (women)<br><br>- IG: 25<br>- CG: 23 usual leisure activities	9 months<br><br>2 sessions of 60 minutes of physical exercise per week, supervised by a trainer.<br>F: start and end	- Psychological symptoms<br><br>- Depression<br><br>- Burden	- S Yesavage Geriatric Depression (α=0.80)<br><br>- Symptom Checklist 90 (α=NR)<br><br>- S Zarit (α=NR)	80% C completed 90% of I exercise sessions.<br><br>IG: no differences in depression or subjective load at the start of I.<br>At the end decreased depression and load in IG.
Tawfik et al. (2021) [22]	RCT	Psychoeducational programme to reduce perceived burden and increase quality of life	70 C<br><br>(60% wives and 40% daughters)<br><br>- IG: 35<br><br>- CG: 35 usual hospital care	2 months<br><br>8 group sessions (15-20 C) of 1h, 1x per week<br><br>F: before and after I	- Burden<br>- Quality of life	- S Zarit (α=NR)<br>- Quality of Life Questionnaire (α=NR)	IG: decreased burden and improved quality of life, as I provided knowledge about dementia and methods to meet demands.<br>CG: increased burden, reflecting need for interventions.

			Drop-outs: 5 IG and 5 CG				
Töpfer et al. (2021) [23]	RCT	I. cognitive-behavioural telephone	51 C<br>- IG: 29<br>- CG: 22	6 months<br><br>12 sessions<br>S: 2.5 years later	-Burden<br><br>- Quality of life<br><br>- Social relations	- S Depression Centre for Epidemiological Studies (α=0.89)<br><br>- Giessen Subjective Complaints List (α=0.94)<br><br>- S Health (physical α=0.83, psychological α=0.81, quality of life α=83)	IG: lower burden and higher quality of life in social relations. Dampens detrimental effects of care on burden and long-term relationships.
Hepburn et al. (2022) [24]	RCT	Synchronous/asynchronous online psychoeducation programme	261 C<br><br>- IG1: 96<br><br>- CG1: 111 usual Community care	43 days<br><br>7 sessions of 75-90 minutes	- Psychological well-being<br><br>- Burden	- S Perceived Stress Cohen (α=0.88)<br><br>- S Depression Centre Epidemiological Studies (α=0.90)	IG: over 60% improved stress and depression from baseline to 3 and 6 months. CG1 little

			- CG2: 54 waiting list	weekly synchronous and 36 asynchronous sessions per week<br>F: baseline and at 3, 6, 9, 9 and 12 months Completed I 14 IG C, 15 CG2 and 8 CG1.	- Stress<br>- Depression<br>- Anxiety	- State-Trait Anxiety Inventory (α=0.95)<br>- S Zarit (α=0.90)<br>- S Pearlin Domain (α=0.76)	improvement and CG2 worsened.<br>IG: decreased anxiety from baseline to 3 and 6 months.<br>I no comparable results on burden, although decreased and improved in IG more than in CG1, no change in CG2.<br>IG: decreased reactions to behaviours, at 3 and 6 months.
Salehinejad et al. (2022) [25]	RCT	I. of web-based health information	50 C<br>- IG: access to web information<br>- CG: access to regular information	8 weeks<br>12 sessions designed<br>F: start and after 2 months	-Knowledge<br>- Attention load<br>- Attitudes	- S Zarit (α=NR)<br>- S Knowledge of Alzheimer's disease (α=NR)<br>- Attitude Questionnaire	IG: improvements in knowledge, burden and attitudes, compared to CG.

Rodríguez et al. (2023) [26]	RCT, pilot	I. technology	53 C<br>- IG: 26<br>- CG: 27	6 months<br><br>F: onset, at 3 and 6 months<br>Dropouts: 3 IG and 5 CG	- Psychological symptoms<br><br>- Charge	- S Usability Simplified System (α=NR)<br><br>- Questionnaire Intention Behaviour	Usability of application by C at 6 months was 72.5% 'good to excellent'.<br><br>No differences between groups, but in IG reduced burden increases with months. Psychological symptoms reduced from baseline and onwards.

Note: The following abbreviations are used:randomised controlled trial (RCT), intervención (I), control group (CG), intervention group (IG), caregivers (C), follow-up(S) and scale (S).

Table S3. Search strategy.

The selection process was carried out as follows: 1) The search string with the described descriptors and filters was executed; 2) The Mendeley reference manager was used to store documents and references, facilitating the removal of duplicate articles; 3) The COVIDENCE tool was utilized for review and data extraction to ensure inter-rater reliability; and 4) The extracted data was stored in an Excel spreadsheet specifically designed for this purpose. This information is included in Table 2.

The search strategy across databases generated a total of 636 results: 228 from Web of Science, 93 from Scopus, 205 from ProQuest, and 110 from PubMed. After an initial screening in Mendeley, where duplicates (n=248) and articles from 2024 (n=3) were removed, a pre-selection was conducted in COVIDENCE by reviewing titles and abstracts. Articles were excluded: (n=58) systematic reviews, literature reviews, integrative reviews, scoping reviews, and meta-analyses; (n=55) studies focusing on other factors of caregivers such as self-care, satisfaction, training, empathy, sleep quality, acceptance, grief, affective skills...; (n=85) analytical, descriptive, correlational, explanatory, cohort, cross-sectional, and comparative studies; (n=6) not relevant to the study objective; and (n=52) protocols, trials, projects, or proposals. Subsequently, a more detailed analysis of these articles resulted in further exclusions: (n=25) interventions conducted with patients instead of caregivers; (n=15) interventions conducted with both patients and caregivers together; (n=42) studies lacking a control group; (n=2) unfinished research; (n=5) inaccessible studies; (n=3) intentional non-random sampling; and (n=1) interventions with fewer than 40 participants. Thus, 28 articles were deemed suitable. The analysis and selection process to determine article suitability for the scoping review was conducted independently by two researchers using COVIDENCE, acting as blind evaluators. Disagreements regarding article inclusion were resolved through consensus between the researchers upon re-evaluation. Ultimately, 26 articles met the criteria and were included in the scoping review. Figure 1 provides a schematic representation of the search process and the final number of articles studied.

Table S4. Preferred Reporting Items for Systematic reviews and Meta-Analyses extension for Scoping Reviews (PRISMA-ScR) Checklist.

Section	Item	PRISMA-ScR checklist item	Reported on page
		**TITLE**	
Title	1	Identify the report as a scoping review.	1
		**ABSTRACT**	
Structured summary	2	Provide a structured summary that includes (as applicable): background, objectives, eligibility criteria, sources of evidence, charting methods, results, and conclusions that relate to the review questions and objectives.	1
		**INTRODUCTION**	
Rationale	3	Describe the rationale for the review in the context of what is already known. Explain why the review questions/objectives lend themselves to a scoping review approach.	1-3
Objectives	4	Provide an explicit statement of the questions and objectives being addressed with reference to their key elements (e.g., population or participants, concepts, and context) or other relevant key elements used to conceptualize the review questions and/or objectives.	3
		**METHODS**	
Protocol and registration	5	Indicate whether a review protocol exists; state if and where it can be accessed (e.g., a Web address); and if available, provide registration information, including the registration number.	3
Eligibility criteria	6	Specify characteristics of the sources of evidence used as eligibility criteria (e.g., years considered, language, and publication status), and provide a rationale.	3-4
Information sources	7	Describe all information sources in the search (e.g., databases with dates of coverage and contact with authors to identify additional sources), as well as the date the most recent search was executed.	4
Search	8	Present the full electronic search strategy for at least 1 database, including any limits used, such that it could be repeated.	4-5
Selection of sources of evidence	9	State the process for selecting sources of evidence (i.e., screening and eligibility) included in the scoping review.	5
Data charting process	10	Describe the methods of charting data from the included sources of evidence (e.g., calibrated forms or forms that have been tested by the team before their use, and whether data charting was done independently or in duplicate) and any processes for obtaining and confirming data from investigators.	5
Data items	11	List and define all variables for which data were sought and any assumptions and simplifications made.	5
Critical appraisal of individual sources of evidence	12	If done, provide a rationale for conducting a critical appraisal of included sources of evidence; describe the methods used and how this information was used in any data synthesis (if appropriate).	N.A.
Synthesis of results	13	Describe the methods of handling and summarizing the data that were charted.	5
**RESULTS**			
Selection of sources of evidence	14	Give numbers of sources of evidence screened, assessed for eligibility, and included in the review, with reasons for exclusions at each stage, ideally using a flow diagram.	4-5, Supplementary Image 1
Characteristics of sources of evidence	15	For each source of evidence, present characteristics for which data were charted and provide the citations.	5

Section	Item	PRISMA-ScR checklist item	Reported on page
Critical appraisal within sources of evidence	16	If done, present data on critical appraisal of included sources of evidence (see item 12).	N.A.
Results of individual sources of evidence	17	For each included source of evidence, present the relevant data that were charted that relate to the review questions and objectives.	Supplementary material (Table 2)
Synthesis of results	18	Summarize and/or present the charting results as they relate to the review questions and objectives.	6-12
**DISCUSSION**			
Summary of evidence	19	Summarize the main results (including an overview of concepts, themes, and types of evidence available), link to the review questions and objectives, and consider the relevance to key groups.	12-14
Limitations	20	Discuss the limitations of the scoping review process.	13-14
Conclusions	21	Provide a general interpretation of the results with respect to the review questions and objectives, as well as potential implications and/or next steps.	14
**FUNDING**			
Funding	22	Describe sources of funding for the included sources of evidence, as well as sources of funding for the scoping review. Describe the role of the funders of the scoping review.	14

Note. N.A.: Not applicable.

Reference

1. Chang, B.L. Cognitive-behavioral intervention for homebound caregivers of persons with dementia. *Nurs. Res.* **1999**, *48*, 173–182. https://doi.org/10.1097/00006199-199905000-00007.
2. Sanford, M.D.; Czaja, S.J.; Martinovich, Z.; Carol, R.N.; Donna, M.S.W.; Schulz, R. E-Care: A telecommunications technology intervention for family caregivers of dementia patients. *Am. J. Geriatr. Psychiatry* **2007**, *15*, 443–448. https://doi.org/10.1097/JGP.0b013e3180437d87.
3. Martín-Carrasco, M.; Domínguez-Panchón, A.I.; González-Fraile, E.; Muñoz-Hermoso, P.; Ballesteros, J. Effectiveness of a psychoeducational intervention group program in the reduction of the burden experienced by caregivers of patients with dementia. EDUCA II Randomized Trial. *Alzheimer Dis. Assoc. Disord.* **2014**, *28*, 79–87. https://doi.org/10.1097/WAD.0000000000000003.
4. Tanner, J.A.; Black, B.S.; Johnston, D.; Hess, E.; Leoutsakos, J.M.; Gitlin, L.N.; Rabins, P.V.; Lyketsos, C.G.; Samus, Q.M. A randomized controlled trial of a community-based dementia care coordination intervention: Effects of MIND at home on caregiver outcomes. *Am. J. Geriatr. Psychiatry* **2015**, *23*, 391–402. https://doi.org/10.1016/j.jagp.2014.08.002.
5. Brown, K.W.; Coogle, C.L.; Wegelin, J. A pilot randomized controlled trial of mindfulness-based stress reduction for caregivers of family members with dementia. *Aging Ment. Health* **2016**, *20*, 1157–1166. https://doi.org/10.1080/13607863.2015.1065790.
6. Cheng, S.T.; Fung, H.H.; Chan, W.C.; Lam, L.C. Short-term effects of a gain-focused reappraisal intervention for dementia caregivers: A double-blind cluster-randomized controlled trial. *Am. J. Geriatr. Psychiatry* **2016**, *24*, 740–750. https://doi.org/10.1016/j.jagp.2016.04.012.
7. Farran, C.J.; Paun, O.; Cothran, F.; Etkin, C.; Rajan, K.B.; Eisenstein, A.; Navaie, M. Impact of an individualized physical activity intervention on improving mental health outcomes in family caregivers of persons with dementia: A randomized controlled trial. *AIMS Med. Sci.* **2016**, *3*, 15–31. https://doi.org/10.3934/medsci.2016.1.15.
8. Hirano, A.; Umegaki, H.; Suzuki, Y.; Hayashi, T.; Kuzuya, M. Effects of leisure activities at home on perceived care burden and the endocrine system of caregivers of dementia patients: A randomized controlled study. *Int. Psychogeriatr.* **2016**, *28*, 261–268. https://doi.org/10.1017/S1041610215001295.
9. Sepe-Monti, M.; Vanacore, N.; Bartorelli, L.; Tognetti, A.; Giubilei, F. The savvy caregiver program: A probe multicenter randomized controlled pilot trial in caregivers of patients affected by Alzheimer’s disease. *J. Alzheimer's Dis.* **2016**, *54*, 1235–1246. https://doi.org/10.3233/JAD-160235.

10. Söylemez, B.A.; Küçükgüçlü, Ö.; Buckwalter, K.C. Application of the progressively lowered stress threshold model with community-based caregivers: A randomized controlled trial. J. Gerontol. Nurs. 2016, 42, 44–54.
11. Berwig, M.; Heinrich, S.; Spahlholz, J.; Hallensleben, N.; Brähler, E.; Gertz, H.J. Individualized support for informal caregivers of people with dementia: Effectiveness of the German adaptation of REACH II. *BMC Geriatr.* **2017**, *17*, 286. https://doi.org/10.1186/s12877-017-0678-y.
12. Mavandadi, S.; Wright, E.M.; Graydon, M.M.; Oslin, D.W.; Wray, L.O. A randomized pilot trial of a telephone-based collaborative care management program for caregivers of individuals with dementia. *Psychol. Serv.* **2017**, *14*, 102–111. https://doi.org/10.1037/ser0000118.
13. Spalding-Wilson, K.N.; Guzmán-Vélez, E.; Angelica, J.; Wiggs, K.; Savransky, A.; Tranel, D. A novel two-day intervention reduces stress in caregivers of persons with dementia. *Alzheimer's Dement. Transl. Res. Clin. Interv.* **2018**, *4*, 450–460. https://doi.org/10.1016/j.trci.2018.08.004.
14. Zwingmann, I.; Hoffmann, W.; Michalowsky, B.; Dreier-Wolfgramm, A.; Hertel, J.; Wucherer, D.; Eichler, T.; Kilimann, I.; Thiel, F.; Teipel, S.; et al. Supporting family dementia caregivers: Testing the efficacy of dementia care management on multifaceted caregivers' burden. *Aging Ment. Health* **2018**, *22*, 889–896. https://doi.org/10.1080/13607863.2017.1399341.
15. Williams, W.; Perkhounkova, Y.; Shaw, C.; Hein, M.; Vidoni, E.; Coleman, C. Supporting family caregivers with technology for dementia home care: A randomized controlled trial. *Innov. Aging* **2019**, *3*, igz037. https://doi.org/10.1093/geroni/igz037.
16. Brewster, G.S.; Epps, F.; Dye, C.E.; Hepburn, K.; Higgins, M.K.; y Parker, M.L. The effect of the "great village" on psychological outcomes, burden, and mastery in African American caregivers of persons living with dementia. *J. Appl. Gerontol.* **2020**, *39*, 1059–1068. https://doi.org/10.1177/0733464819874574.
17. Szczepańska-Gieracha, J., Jaworska-Burzyńska, L., Boroń-Krupińska, K. y Kowalska, J. (2020). Nonpharmacological forms of therapy to reduce the burden on caregivers of patients with dementia: A pilot intervention study. International Journal of Environmental Research and Public Health, 17(24), 9153. https://doi.org/10.3390/ijerph17249153.
18. Terracciano, A.; Artese, A.; Yeh, J.; Edgerton, L.; Granville, L.; Aschwanden, D.; Luchetti, M.; Glueckauf, R.; Stephan, Y.; Sutin, A.; et al. Effectiveness of powerful tools for caregivers on caregiver burden and on care recipient behavioral and psychological symptoms of dementia: A randomized controlled trial. *J. Am. Med. Dir. Assoc.* **2020**, *21*, 1121–1127. https://doi.org/10.1016/j.jamda.2019.11.011.
19. Bravo-Benítez, J.; Cruz-Quintana, F.; Fernández-Alcántara, M.; Pérez-Marfil, M.N. Intervention program to improve grief-related symptoms in caregivers of patients diagnosed with dementia. *Front. Psychol.* **2021**, *12*, 628750. https://doi.org/10.3389/fpsyg.2021.628750.
20. Hives, B.; Buckler, J.; Weiss, J.; Schilf, S.; Johansen, K.; Epel, E.; Puterman, E. The effects of aerobic exercise on psychological functioning in family caregivers: Secondary analyses of a randomized controlled trial. *Ann. Behav. Med.* **2021**, *55*, 65–76. https://doi.org/10.1093/abm/kaaa031.
21. Madruga, M.; Gozalo, M.; Prieto, J.; Rohlfs Domínguez, P.; Gusi, N. Effects of a home-based exercise program on mental health for caregivers of relatives with dementia: A randomized controlled trial. *Int. Psychogeriatr.* **2021**, *33*, 359–372. https://doi.org/10.1017/S104161022000157X.
22. Tawfik, N.M.; Sabry, N.A.; Darwish, H.; Mowafy, M.; Soliman, S. Psychoeducational program for the family member caregivers of people with dementia to reduce perceived burden and increase patient's quality of life: A randomized controlled trial. *J. Prim. Care Community Health* **2021**, *12*, 1-7. https://doi.org/10.1177/21501327211014088.
23. Töpfer, N.F.; Sittler, M.C.; Lechner-Meichsner, F.; Theurer, C.; Wilz, G. Long-term effects of telephone-based cognitive-behavioral intervention for family caregivers of people with dementia: Findings at 3-year follow-up. *J. Consult. Clin. Psychol.* **2021**, *89*, 341–349. https://doi.org/10.1037/ccp0000640.
24. Hepburn, K.; Nocera, J.; Higgins, M.; Epps, F.; Brewster, G.; Lindauer, A.; Morhardt, D.; Shah, R.; Bonds, K.; Nash, R.; et al. Results of a randomized trial testing the efficacy of Tele-Savvy, an online synchronous/asynchronous psychoeducation program for family caregivers of persons living with dementia. *Gerontologist* **2022**, *62*, 616–628. https://doi.org/10.1093/geront/gnab029.
25. Salehinejad, S.; Jannati, N.; Azami, M.; Mirzaee, M.; y Bahaadinbeigy, K. A web-based information intervention for family caregivers of patients with Dementia: A randomized controlled trial. *J. Inf. Sci.* **2022**, *50*, 104–115. https://doi.org/10.1177/01655515221081353.
26. Rodríguez, M.J.; Martinez Kercher, V.; Jordan, E.J.; Savoy, A.; Hill, J.R.; Werner, N.; Owora, A.; Castelluccio, P.; Boustani, M.A.; Holden, R.J. Technology caregiver intervention for Alzheimer's disease (I-CARE): Feasibility and preliminary efficacy of brain carenotes. *J. Am. Geriatr. Soc.* **2023**, *71*, 3836–3847. https://doi.org/10.1111/jgs.18591.
